# Supplementary material for: Using mobility data in the design of optimal lockdown strategies for the COVID-19 pandemic
Source: PLoS Comput Biol. 2021 Aug 12;17(8):e1009236. doi: 10.1371/journal.pcbi.1009236 (PMC8360388; doi:10.1371/journal.pcbi.1009236)
Supplement: S1 Appendix — Details on computation of the basic reproduction number R(t) and explanation of Approximate Bayesian Computation inferential procedure. (PDF) [file pcbi.1009236.s001.pdf]

S1 Appendix: additional mathematical details
Using mobility data in the design of optimal lockdown strategies for the COVID-19 pandemic

Ritabrata Dutta[1]*, Susana Gomes[2], Dante Kalise[3], Lorenzo Pacchiardi[4]

[1] *Department of Statistics, Warwick University, Coventry, UK*

[2] *Department of Mathematics, Warwick University, Coventry, UK*

[3] *School of Mathematical Sciences, University of Nottingham, Nottingham, UK*

[4] *Department of Statistics, University of Oxford, Oxford, UK*

1 Computation of $\mathcal{R}(t)$

We explain here how to compute the reproduction rate ($\mathcal{R}$) for our model, which is considered as one of the main measures to quantify the spread of an epidemic and the efficiency of our lockdown strategies proposed in the Results and Discussion Section in the main body of the paper, and measures the average number of secondary infections an infected individual is capable of generating in a fully susceptible population. Our computation strategy follows [3]; first, we define the *next generation matrix*, which relates the numbers of newly infected individuals in the various categories in consecutive moments, before and after a contact (in our case, a time step) in the case where one single person interacts with a fully susceptible population. To do this, fix $S_i = N_i$ and consider the Jacobian matrix of the infection subsystem of our model (Eqs. (1)-(8) in the main body) – which is composed by states $\left\{E_i, I_i^{SC_1}, I_i^{SC_2}, I_i^{C_1}, I_i^{C_2}\right\}$, $i = 1, \dots, 5$ (we note that the states R_i and D_i were removed, as they are "final" states: once an individual is in one of these states, they remain there). Using the same notation as in [3], this Jacobian has the following form:

$$\mathbf{J_0} = \begin{bmatrix} -\kappa I & \beta\frac{SC}{N} & \beta\frac{SC}{N} & 0 & 0 \\ \rho\kappa I & -\gamma_C I & 0 & 0 & 0 \\ (1-\rho)\kappa I & 0 & -\gamma_R I & 0 & 0 \\ 0 & \rho'\gamma_C I & 0 & -\nu I & 0 \\ 0 & (1-\rho')\gamma_C I & 0 & 0 & -\gamma_{R,C} I \end{bmatrix},$$

where we note that I is the 5×5 identity matrix, and each zero corresponds to a 5×5 zero matrix. We also slightly abused the notation in the other entries. For example, the matrices $\frac{SC}{N}$, $\rho\kappa I$, etc., are 5×5 matrices whose entries are given by:

$$\left(\frac{SC}{N}\right)_{ij} = \frac{S_i C_{ij}}{N_j}, \qquad (\rho\kappa I)_{ij} = \kappa\rho_i\delta_{ij},$$

where δ_{ij} is the Kronecker delta, and similarly for the other matrices.

The matrix $\mathbf{J_0}$ is decomposed into the *transmission matrix*, T, and the *transition matrix*, Σ, defined as follows:

$$T = \begin{bmatrix} 0 & \beta\frac{SC}{N} & \beta\frac{SC}{N} & 0 & 0 \\ 0 & 0 & 0 & 0 & 0 \\ 0 & 0 & 0 & 0 & 0 \\ 0 & 0 & 0 & 0 & 0 \\ 0 & 0 & 0 & 0 & 0 \end{bmatrix}, \qquad \Sigma = \begin{bmatrix} -\kappa I & 0 & 0 & 0 & 0 \\ \rho\kappa I & -\gamma_C I & 0 & 0 & 0 \\ (1-\rho)\kappa I & 0 & -\gamma_R I & 0 & 0 \\ 0 & \rho'\gamma_C I & 0 & -\nu I & 0 \\ 0 & (1-\rho')\gamma_C & 0 & 0 & -\gamma_{R,C} I \end{bmatrix} = \mathbf{J_0} - T.$$

*Corresponding author: Ritabrata.Dutta@warwick.ac.uk.

The *next generation matrix* is then defined as $K_L = -T\Sigma^{-1}$. The reproduction number $\mathcal{R}$ is the *spectral radius* of this matrix, namely the absolute value of its largest eigenvalue, which represents the secondary number of infections a single infected individual is capable of generating in a fully susceptible population. As T depends on the contact matrix C, the reproduction number will be a function of the Google mobility for different locations ($m^{school}(t)$, $m^{work}(t)$ and $m^{other}(t)$). Hence, we will focus on studying the evolution of $\mathcal{R}(t)$ over time.

2 Details on Approximate Bayesian Computation

Approximate Bayesian computation (ABC) gives an approximation of the posterior distribution of the parameters, starting from a prior $\pi(\theta)$ and a (possible stochastic) simulator model $M(\theta)$, for which the likelihood $p(\mathbf{x}|\theta)$ cannot be computed. Specifically, the true posterior is obtained via Bayes' theorem as

$$\pi(\theta|\mathbf{x}^{obs}) = \frac{\pi(\theta)p(\mathbf{x}^{obs}|\theta)}{p(\mathbf{x}^{obs})}.$$

In ABC, we approximate this expression by looking for the values of the parameters which best approximate the observations. The fundamental ABC rejection sampling scheme iterates the following steps:

- Draw θ from the prior $\pi(\theta)$.
- Simulate a synthetic dataset $\mathbf{x}^{sim}$ from the simulator-based model $M(\theta)$.
- Accept the parameter value θ if $\mathbf{d}(\mathbf{x}^{sim}, \mathbf{x}^{obs}) < \gamma$. Otherwise, reject θ.

Here, the metric on the dataspace $\mathbf{d}(\mathbf{x}^{sim}, \mathbf{x}^{obs})$ measures the closeness between $\mathbf{x}^{sim}$ and $\mathbf{x}^{obs}$. The accepted values of θ are thus sampled from a distribution $\pi_{ABC}(\theta|\mathbf{x}^{obs}) \propto \pi(\theta)p_{\mathbf{d},\gamma}(\mathbf{x}^{obs}|\theta)$, where $p_{\mathbf{d},\gamma}(\mathbf{x}^{obs}|\theta)$ is an approximation to the intractable likelihood function $p(\mathbf{x}^{obs}|\theta)$:

$$p_{\mathbf{d},\gamma}(\mathbf{x}^{obs}|\theta) = \int p(\mathbf{x}^{sim}|\theta)\mathbb{K}_\gamma(\mathbf{d}(\mathbf{x}^{sim}, \mathbf{x}^{obs}))d\mathbf{x}^{sim}.$$

Here, $\mathbb{K}_\gamma(\mathbf{d}(\mathbf{x}^{sim}, \mathbf{x}^{obs}))$ is a probability density function proportional to $\mathbf{1}\{\mathbf{d}(\mathbf{x}^{sim}, \mathbf{x}^{obs}) \leq \gamma\}$, $\mathbf{1}\{\cdot\}$ being an indicator function which equals 1 when the condition in the brackets is true and 0 otherwise.

This guarantees that, in principle, the above approximate likelihood converges to the true one when $\gamma \to 0$. In this paper, we used the PMCABC algorithm [1] as implemented in the Python library ABCpy [2] allowing efficient parallelization using MPI, to perform parameter inference. This is an iterative algorithm considering a set of points $\{\theta_i\}$ which are given a certain weight representing how much the sample x_i generated by each of them is close to the observation. The algorithm proceeds by iteratively perturbing the parameters and performing simulations from the model, and reducing the threshold γ so that the approximation to the posterior distribution improves. At the end of the algorithm, a weighted set of parameter points which are samples from the approximate posterior $\pi_{ABC}(\theta|\mathbf{x}^{obs})$ is returned.

For the sake of calibrating the model, we want to match the number of people in I^C (summed over all age groups) and the daily deaths, by date of reporting, for each of the 5 considered age groups; the model is structured so that it returns those values as outputs. Therefore, we consider the set of variables $\mathbf{x} = ((\Delta D_1(t), \Delta D_2(t), \Delta D_3(t), \Delta D_4(t), \Delta D_5(t))_{t=1}^T, (I^C_{tot}(t))_{t=18}^T)$ where we denote by t the day since the start of the dynamics, by $\Delta D_i(t) = D_i(t) - D_i(t-1)$ the deaths occurring on day t in age group i, and we consider $I^C_{tot}(t) = \sum_{i=1}^5 I^C_i$. The corresponding observation is denoted as:

$$\mathbf{x}^{obs} = ((\Delta D_1^{obs}(t), \Delta D_2^{obs}(t), \Delta D_3^{obs}(t), \Delta D_4^{obs}(t), \Delta D_5^{obs}(t))_{t=1}^T, (I^{C,obs}_{tot}(t))_{t=18}^T).$$

As discussed in the Model Calibration Section in the main body of the paper, data on I^C is available only from the 18th of March, and therefore we discarded the first 17 days for the corresponding observations.

We now define a distance for ABC that enables us to make use of the above data, by relying on a weighted sum of the pointwise Euclidean distances between the different elements of the trajectories. Specifically, let

us denote the pointwise Euclidean distances between the different elements of simulated and observed data by:

$$d_{D,i} = \sum_{t=1}^{T} (\Delta D_i(t) - \Delta D_i^{obs}(t))^2, \quad d_I = \sum_{t=18}^{T} (I_{tot}^{C}(t) - I_{tot}^{C,obs}(t))^2.$$

Using this, the final distance we consider is:

$$d(\mathbf{x}, \mathbf{x}^{obs}) = \sum_{i=1}^{5} d_{D,i} w_{D,i} + w_I d_I,$$

where $w_{D,i}$ and w_I are weights which we can fix according to considerations on the speed of convergence. It is clear that the above, being a combination of Euclidean distances, is a valid distance for $\mathbf{x}$ for each choice of the weight; the latter however are important for the ABC algorithm in practice, as they force the algorithm to constrain more or less on some of the distances. The weights we have found to work best for the problem at hand (as they gave faster convergence) are: $w_D = (1, 1, 1, 2, 2)$ and $w_I = 0.1$.

References

[1] Beaumont MA. Approximate Bayesian computation in evolution and ecology. Annual review of ecology, evolution, and systematics. 2010;41:379–406.

[2] Dutta R, Schoengens M, Onnela JP, Mira A. ABCpy: A user-friendly, extensible, and parallel library for approximate Bayesian computation. In: Proceedings of the Platform for Advanced Scientific Computing Conference; 2017. p. 1–9.

[3] Gatto M, Bertuzzo E, Mari L, Miccoli S, Carraro L, Casagrandi R, et al. Spread and dynamics of the COVID-19 epidemic in Italy: Effects of emergency containment measures. Proceedings of the National Academy of Sciences. 2020;117(19):10484–10491.
