# Supplementary material for: Using mobility data in the design of optimal lockdown strategies for the COVID-19 pandemic
Source: PLoS Comput Biol. 2021 Aug 12;17(8):e1009236. doi: 10.1371/journal.pcbi.1009236 (PMC8360388; doi:10.1371/journal.pcbi.1009236)
Supplement: S2 Appendix — Inference results on the posterior distribution of parameters and on the evolution of the unobserved compartments, using data until the 23rd of May; additional optimal control results using data until 11th May. (PDF) [file pcbi.1009236.s002.pdf]

S2 Appendix: additional experimental results
Using mobility data in the design of optimal lockdown strategies for the COVID-19 pandemic

Ritabrata Dutta[1]*, Susana Gomes[2], Dante Kalise[3], Lorenzo Pacchiardi[4]

[1] *Department of Statistics, Warwick University, Coventry, UK*

[2] *Department of Mathematics, Warwick University, Coventry, UK*

[3] *School of Mathematical Sciences, University of Nottingham, Nottingham, UK*

[4] *Department of Statistics, University of Oxford, Oxford, UK*

1 Proof of concept: England data until 23rd May

1.1 Inferred parameters

We calibrate our model on data up to four different days representing different stages of the epidemic (11th of April on the peak of the epidemic and further towards the tail, up to the 23rd of May, as shown in Fig 1). The amount of available data over those four days affects how well our model was able to fit the data and predict the future. We report the posterior mean and the corresponding standard deviation for each model parameter and for each observation horizon t_{obs} in Table. 1. We refer to Sections 2.1 and 2.2 in the main body for the definition of model parameters. We notice that the estimated values differ slightly over the

Figure 1: **Evolution of the epidemics in England, together with ending dates of the observation period t_{obs} used in this study.** The red line represents the number of hospitalized people (scale on y axis on the left), while the blue line represents the daily number of deaths (scale on the right).

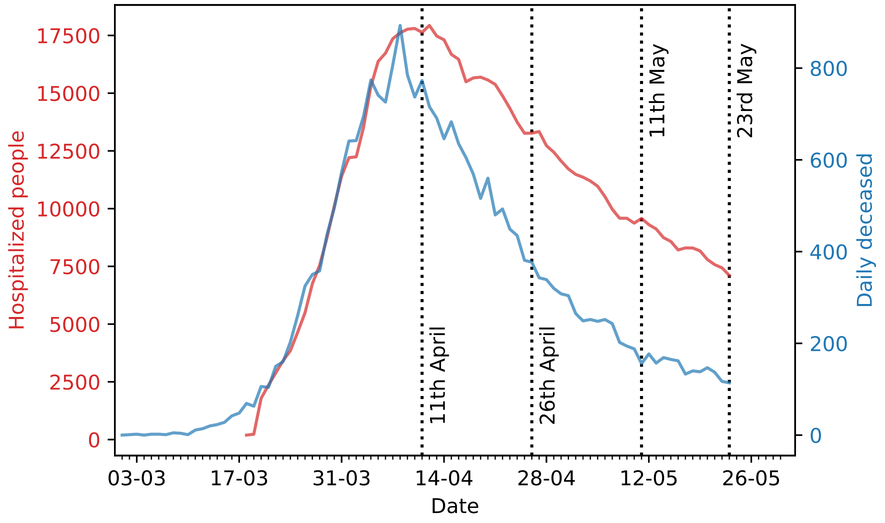

*Corresponding author: Ritabrata.Dutta@warwick.ac.uk.

days, but they are quite similar on 11th and 23rd of May as in those days we do not observe any significant changes in the dynamics of the epidemics.

As point estimates are not able to capture the correlations present between parameters, we report the inferred joint posterior distributions using data until 23rd May between pairs of parameters in Figures 2, 3 and 4. The posterior distribution is obtained by Kernel Density Estimate (KDE) on the set of posterior samples.

Table 1: **Estimated posterior mean and standard deviation of model parameters for England,** using the different horizons for fitting the model. We note the large standard deviation for the ρ and ρ' parameters related to younger age groups, with respect to the older age groups. This is due to the fact that less information is available with regards to the severity of infection for those age groups, thus rendering the estimate harder. This also proves the ability of our technique to assign meaningful uncertainty ranges. We also point out that the estimated standard deviation is larger than the posterior mean for some of the parameters; this is due to the fact that the posterior distribution is not centered on the posterior mean but skewed.

**Observation period**	d_L	d_C	d_R	$d_{R,C}$	d_D
**1st March-11th Apr**	3.09 ± 1.78	4.12 ± 2.16	2.90 ± 1.92	9.94 ± 2.67	4.98 ± 2.35
**1st March-26th Apr**	1.61 ± 0.51	2.46 ± 1.12	1.67 ± 0.50	11.42 ± 1.83	5.19 ± 2.33
**1st March-11th May**	1.50 ± 0.43	2.24 ± 0.87	1.81 ± 0.68	11.95 ± 1.60	5.83 ± 2.14
**1st March-23rd May**	1.57 ± 0.42	2.12 ± 0.80	1.54 ± 0.40	12.08 ± 1.51	5.54 ± 2.19
**1st March-31st Aug**	1.81 ± 0.50	2.84 ± 1.08	1.74 ± 0.49	12.65 ± 1.25	7.44 ± 1.80
**Observation period**	β	α_{123}	α_4	α_5	N^{in}
**1st March-11th Apr**	0.13 ± 0.06	0.48 ± 0.15	0.39 ± 0.26	0.68 ± 0.23	303 ± 132
**1st March-26th Apr**	0.13 ± 0.03	0.64 ± 0.18	0.50 ± 0.25	0.74 ± 0.19	249 ± 140
**1st March-11th May**	0.12 ± 0.03	0.59 ± 0.19	0.54 ± 0.27	0.75 ± 0.20	264 ± 131
**1st March-23rd May**	0.13 ± 0.03	0.63 ± 0.21	0.57 ± 0.23	0.71 ± 0.23	276 ± 133
**1st March-31st Aug**	0.13 ± 0.03	0.36 ± 0.06	0.43 ± 0.28	0.74 ± 0.19	340 ± 111
**Observation period**	ρ_1	ρ_2	ρ_3	ρ_4	ρ_5
**1st March-11th Apr**	0.08 ± 0.07	0.11 ± 0.12	0.16 ± 0.16	0.52 ± 0.25	0.81 ± 0.14
**1st March-26th Apr**	0.08 ± 0.07	0.10 ± 0.12	0.10 ± 0.08	0.51 ± 0.21	0.82 ± 0.13
**1st March-11th May**	0.07 ± 0.07	0.04 ± 0.04	0.09 ± 0.08	0.59 ± 0.23	0.83 ± 0.13
**1st March-23rd May**	0.06 ± 0.06	0.05 ± 0.05	0.08 ± 0.08	0.54 ± 0.22	0.79 ± 0.14
**1st March-31st Aug**	0.07 ± 0.07	0.05 ± 0.05	0.12 ± 0.14	0.53 ± 0.21	0.81 ± 0.14
**Observation period**	ρ'_1	ρ'_2	ρ'_3	ρ'_4	ρ'_5
**1st March-11th Apr**	0.28 ± 0.26	0.21 ± 0.23	0.26 ± 0.26	0.42 ± 0.22	0.82 ± 0.12
**1st March-26th Apr**	0.28 ± 0.23	0.22 ± 0.23	0.33 ± 0.26	0.35 ± 0.16	0.80 ± 0.13
**1st March-11th May**	0.29 ± 0.26	0.35 ± 0.32	0.31 ± 0.24	0.29 ± 0.14	0.78 ± 0.14
**1st March-23rd May**	0.26 ± 0.23	0.28 ± 0.25	0.33 ± 0.27	0.26 ± 0.11	0.80 ± 0.13
**1st March-31st Aug**	0.33 ± 0.27	0.30 ± 0.27	0.33 ± 0.27	0.35 ± 0.18	0.81 ± 0.14

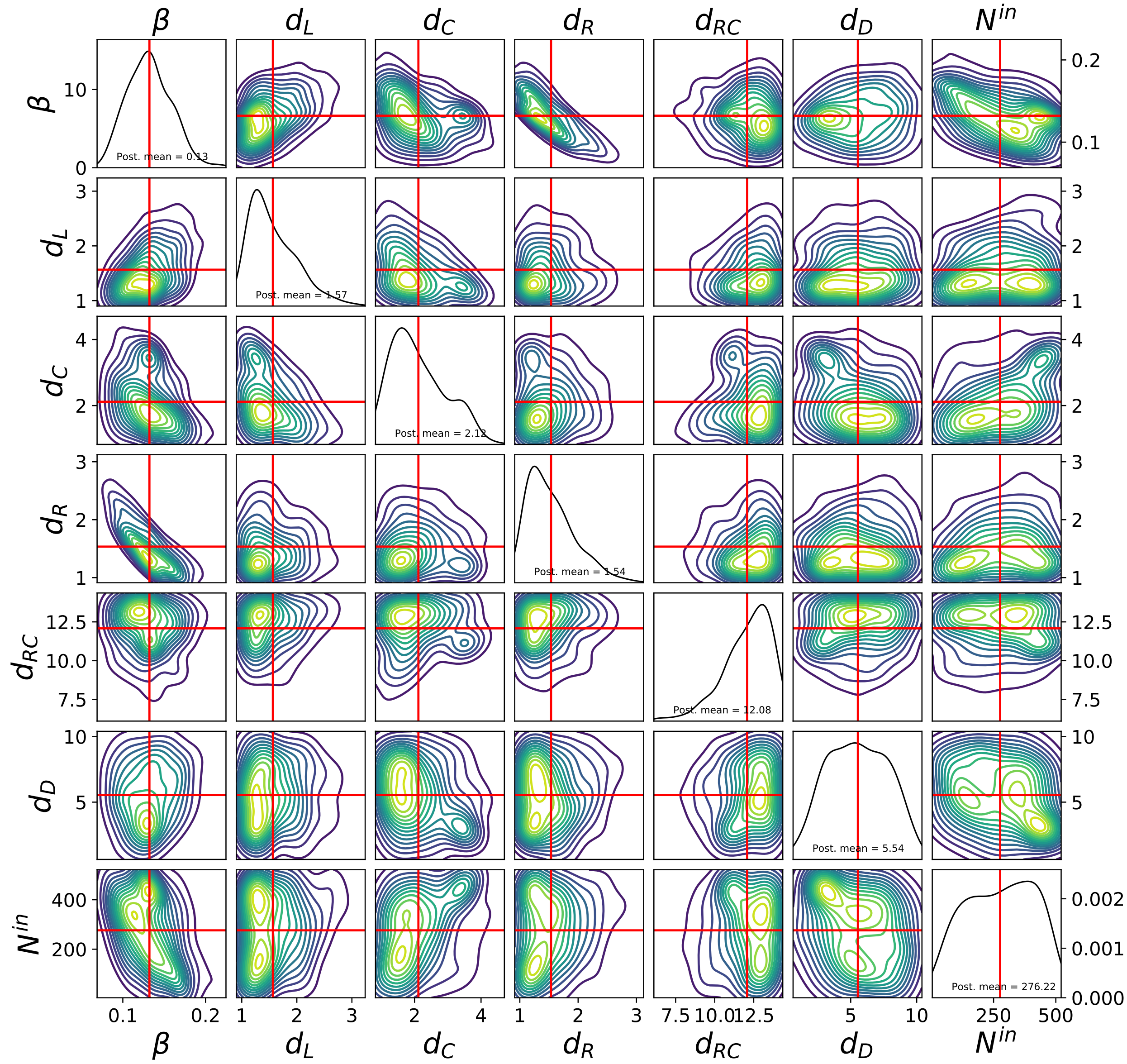

Figure 2: **Single and bivariate marginals (represented using contour plots) for the parameters describing the transition between states for England, obtained using data until 23rd May**; note for instance the negative correlation between β and d_R; this is expected from the dynamics of the model (the longer a person stay in the infectious state, the more people can infect; therefore, a similar dynamics can be obtained by a lower value of β).

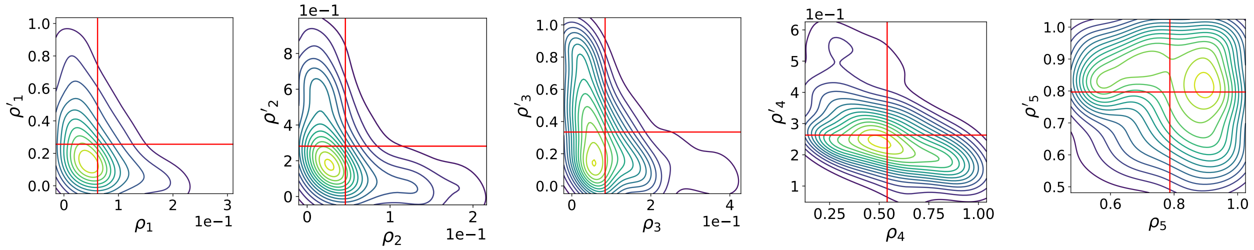

Figure 3: **Contour plots representing bivariate posterior plots for ρ_i and ρ'_i, for all age groups ($i = 1, \dots, 5$) for England, obtained using data until 23rd May**. For all of them, some negative correlation is present; this is very evident in the case of age group 4.

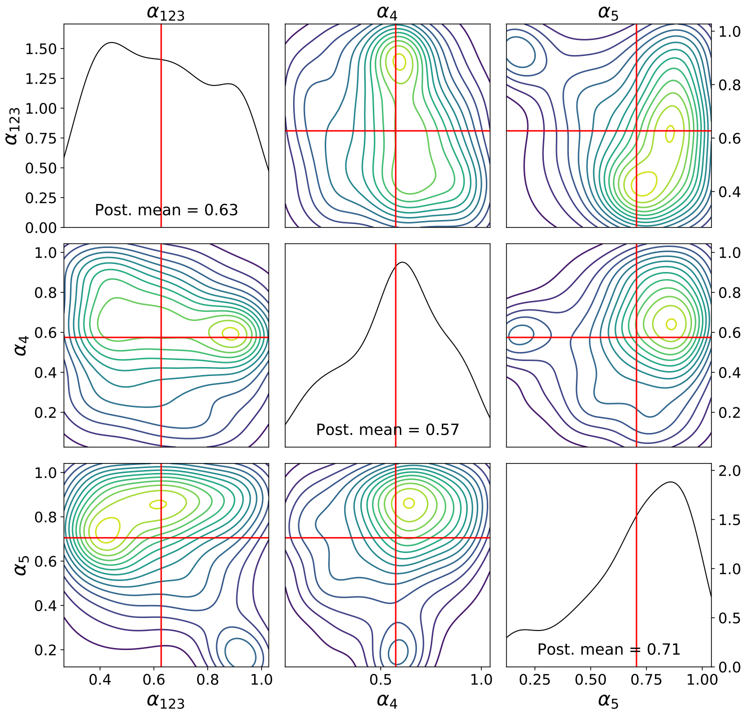

Figure 4: **Single and bivariate marginals (represented using contour plots) for the parameters connecting the reduction in mobility to the change of social contacts for England, obtained using data until 23rd May**.

1.2 Evolution of the epidemics predicted by our model

As devising a successful lockdown strategy depends on the accuracy of the prediction of our model, we compare the predicted number (median prediction and 99 percentile credibility interval) of hospitalized people and daily deaths of our calibrated model with real data on the four observation horizons considered here. To do this, we integrate the dynamical model using i.i.d. posterior samples from the ABC posterior distribution on each of these days and we show our results in Fig 5. In this way we are able to highlight how providing additional information to the model changes its predictions for the future.

Additionally, we provide the estimated basic reproduction number $\mathcal{R}$ (Section 2 of Supporting Information S1) from the dynamics obtained from the posterior sample points, and plot that with the relative credibility bands. We note that, with the exception of the model calibrated on data until the 11th of April, the estimated reproduction number has a value larger than 2 before the introduction of containment measures by the government, which then decreases below 1 a few days afterwards, and remaining below one until the end of the training period. We also note that towards the end of the training period, the credibility interval includes some values above 1, due to the fact that the values of people's mobility have increased in later weeks. Finally, plots comparing the daily number of deaths with real data stratified by age groups are reported in Sections 1.3.

The results presented in Fig 5 highlight that it is hard to forecast precisely the evolution of the epidemic with a simple compartmental model as the one we consider. This can be seen from the fact that using different observation horizons to determine the parameters of the model leads to very different predictions of the evolution; this phenomenon is extremely evident in the first line of Fig 5 (i.e. using data until the 11th of April), where the predicted number of deaths and hospitalized people is much larger than what eventually turned out to be the case (we remark that the prediction is taking into account the measured mobility even throughout the prediction horizon). We also remark that our model systematically overestimates the number of deceased in the tail of the epidemics; this could probably be explained by the fact that it does not take into account the increased capacity of the health system to fight the disease.

1.3 Deaths for each age group

In Figure 6 we report the median and 99 percentile credibility interval of the daily deceased in each of the 5 age groups, and we compare it to the actual data (green). Note that the credibility interval is larger for age groups 1 and 2; this is expected, as the number of deaths in that age groups is relatively small, so that achieving a good fit is harder. Moreover, we also report the cumulative deaths over the different age groups in Figure 7.

See https://github.com/OptimalLockdown/MobilitySEIRD-England for additional plots with regards to the unobserved compartments.

Figure 5: **Comparison of predictions of our model with the real number of hospitalized people with COVID-19 and total daily deaths (green), for England.** The solid red line denotes the median prediction, filled spaces denote the 99% credible interval and the vertical dashed line denotes the observation horizon. The different rows represent different observation horizons, while the columns represent number of people in hospital (I^C compartment, left column), daily deceased (middle column) and value of $\mathcal{R}(t)$ (right column).

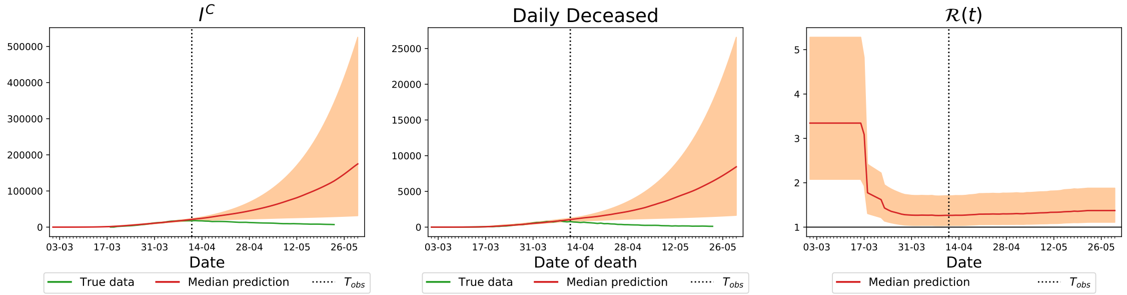

(a) 11th of April

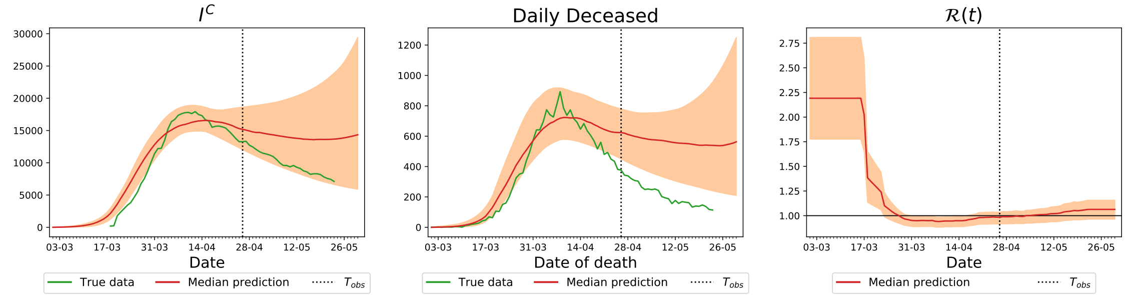

(b) 26th of April

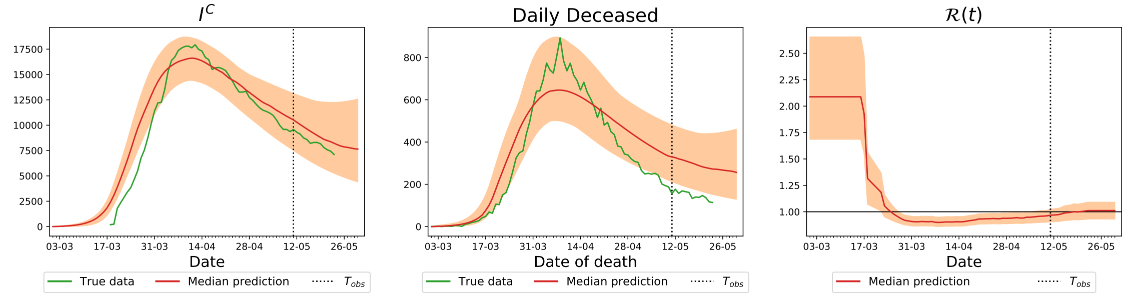

(c) 11th of May

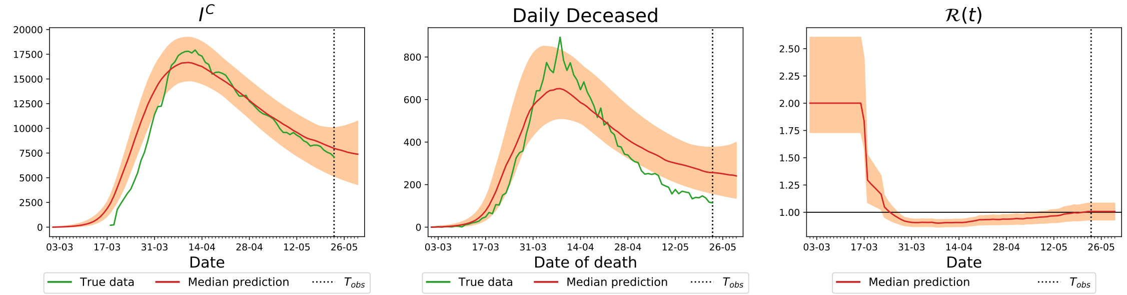

(d) 23rd of May

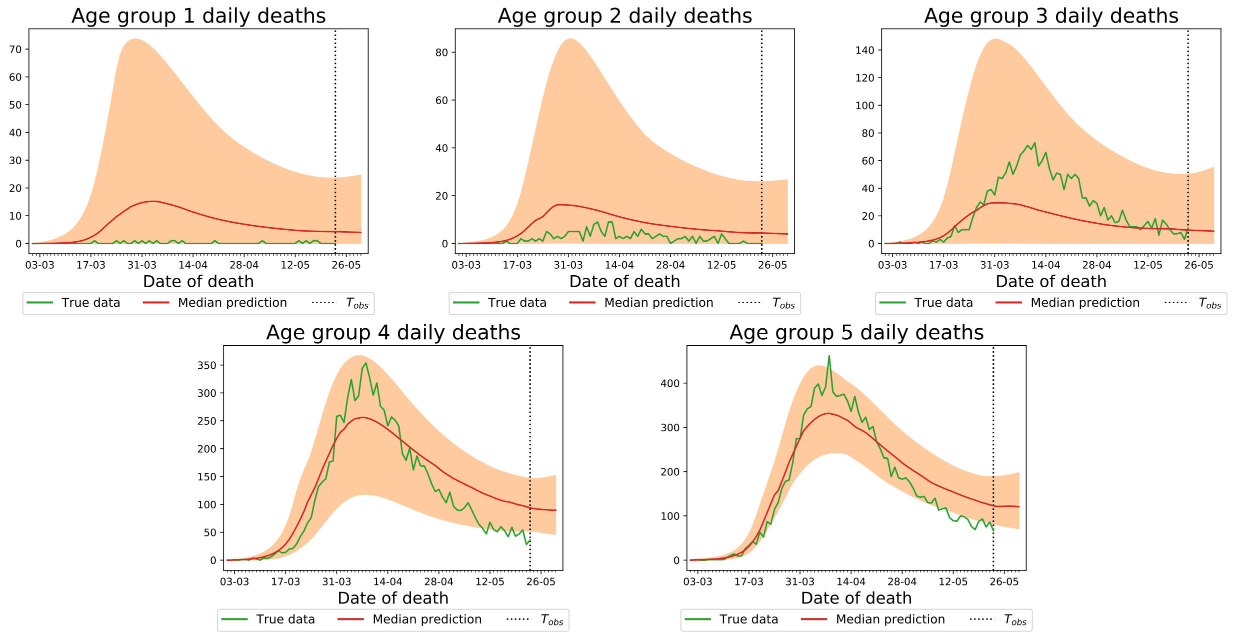

Figure 6: **Comparison of number of daily deceased predicted by our model and actual one (green line), for each age group for England, obtained using data until 23rd May**.

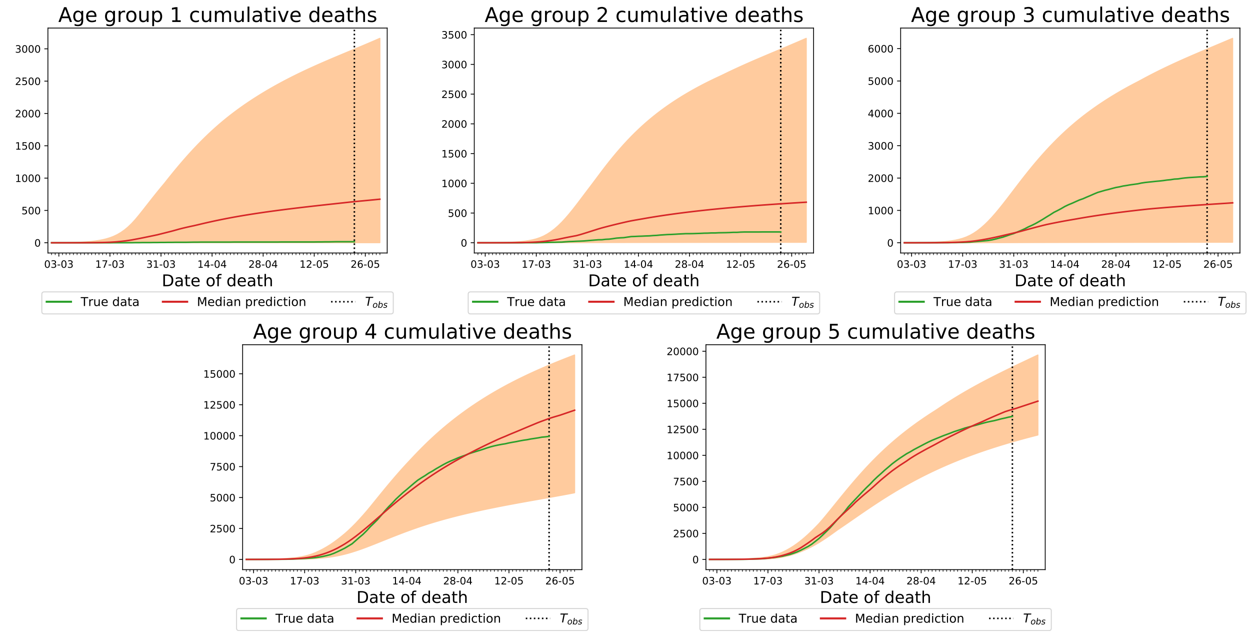

Figure 7: **Comparison of cumulative deaths predicted by our model and actual one (green line), for each age group for England, obtained using data until 23rd May**. Note that the number of deaths in Age Group 1 is very much overestimated by our model. This is due to the relatively small number of deaths in that age group, which make it hard to obtain a good fit.

2 Study on England data until 31st August

2.1 Inferred parameters

We now present results on the case study using data for England until 31st August. The posterior mean and standard deviation of model parameters is reported in Table 1. In Figures 8, 9 and 10 we instead provide posterior plots as above.

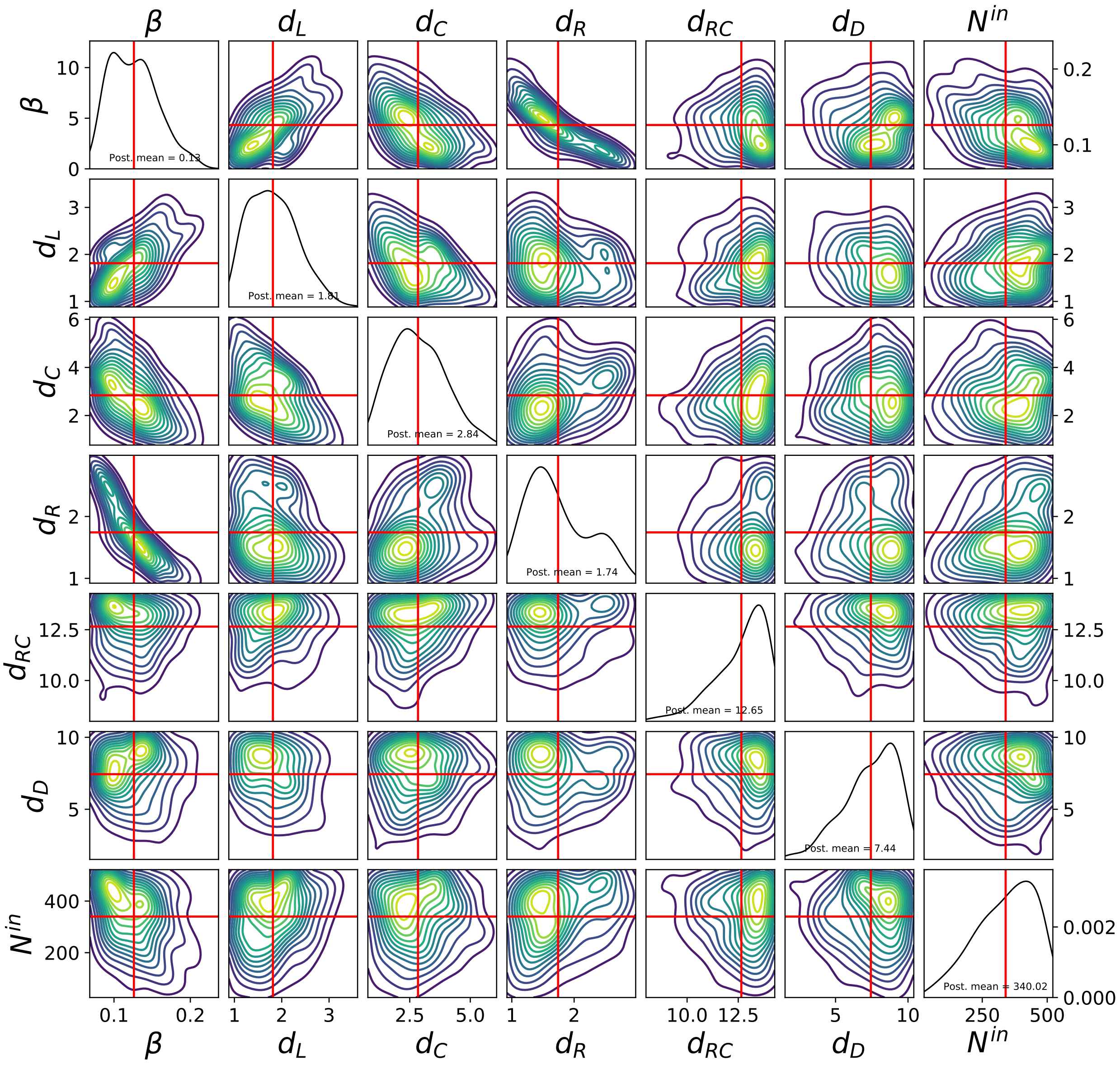

Figure 8: **Single and bivariate marginals (represented using contour plots) for the parameters describing the transition between states for England, obtained using data until 31st August**; note for instance the negative correlation between β and d_R; this is expected from the dynamics of the model (the longer a person stay in the infectious state, the more people can infect; therefore, a similar dynamics can be obtained by a lower value of β).

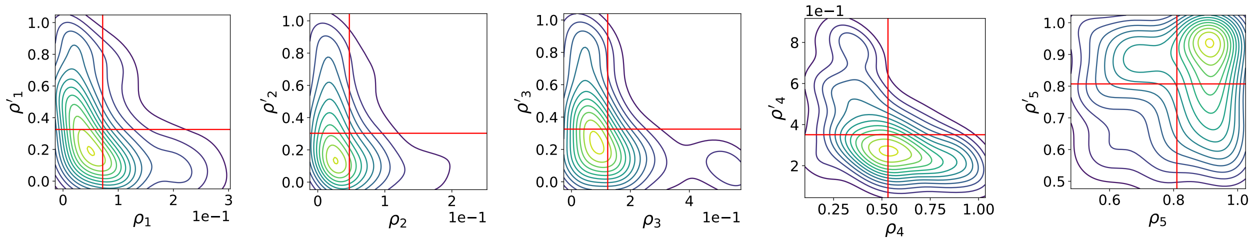

Figure 9: **Contour plots representing bivariate posterior plots for ρ_i and ρ'_i, for all age groups ($i = 1, \ldots, 5$) for England, obtained using data until 31st August**. For all of them, some negative correlation is present; this is very evident in the case of age group 4.

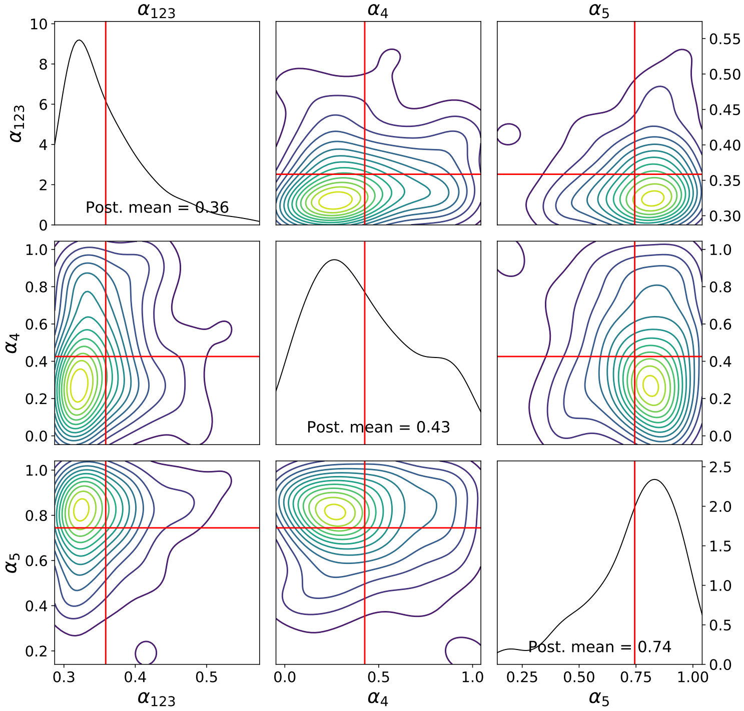

Figure 10: **Single and bivariate marginals (represented using contour plots) for the parameters connecting the reduction in mobility to the change of social contacts for England, obtained using data until 31st August**.

2.2 Deaths for each age group

The main body of the paper reports the predicted evolution of number of hospitalized people and daily deceased, as well as the estimated evolution of $\mathcal{R}(t)$, using data for England until 31st of August, and compares that with the observation. Here, in Figure 11, we report the median and 99 percentile credibility interval of the daily deceased in each of the 5 age groups for the same time interval, and we compare it to the actual data (green). Note that the credibility interval is larger for age groups 1 and 2; this is expected, as the number of deaths in that age groups is relatively small, so that achieving a good fit is harder.

See https://github.com/OptimalLockdown/MobilitySEIRD-England for additional plots with regards to the unobserved compartments.

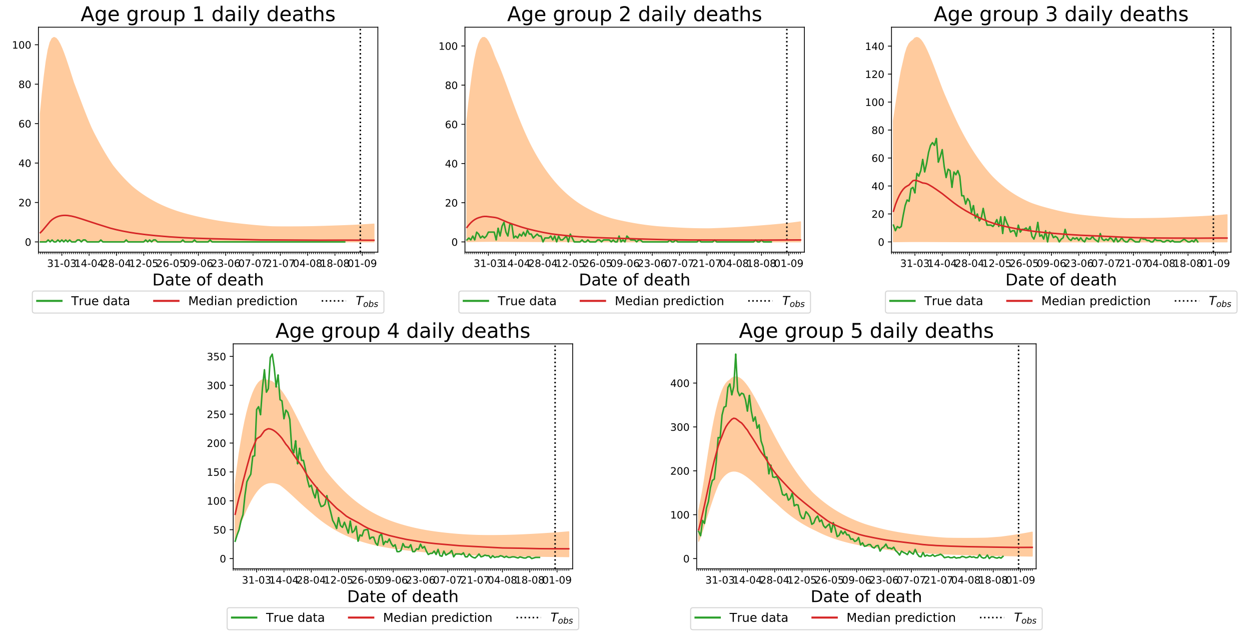

Figure 11: **Comparison of number of daily deceased predicted by our model and actual one (green line), for each age group for England, obtained using data until 31st August**.

3 Study on France data until 31st August

3.1 Inferred parameters

We now present results on the case study using data for England until 31st August. The posterior mean and standard deviation of model parameters is reported in Table 2. In Figures 12, 13 and 14 we instead provide posterior plots as above.

3.2 Deaths for each age group

The main body of the paper reports the predicted evolution of number of hospitalized people and daily deceased, as well as the estimated evolution of $\mathcal{R}(t)$, using data for England until 31st of August, and compares that with the observation. Here, in Figure 15, we report the median and 99 percentile credibility interval of the daily deceased in each of the 5 age groups for the same time interval, and we compare it to the actual data (green). Note that the credibility interval is larger for age groups 1 and 2; this is expected, as the number of deaths in that age groups is relatively small, so that achieving a good fit is harder.

See https://github.com/OptimalLockdown/MobilitySEIRD-France for additional plots with regards to the unobserved compartments.

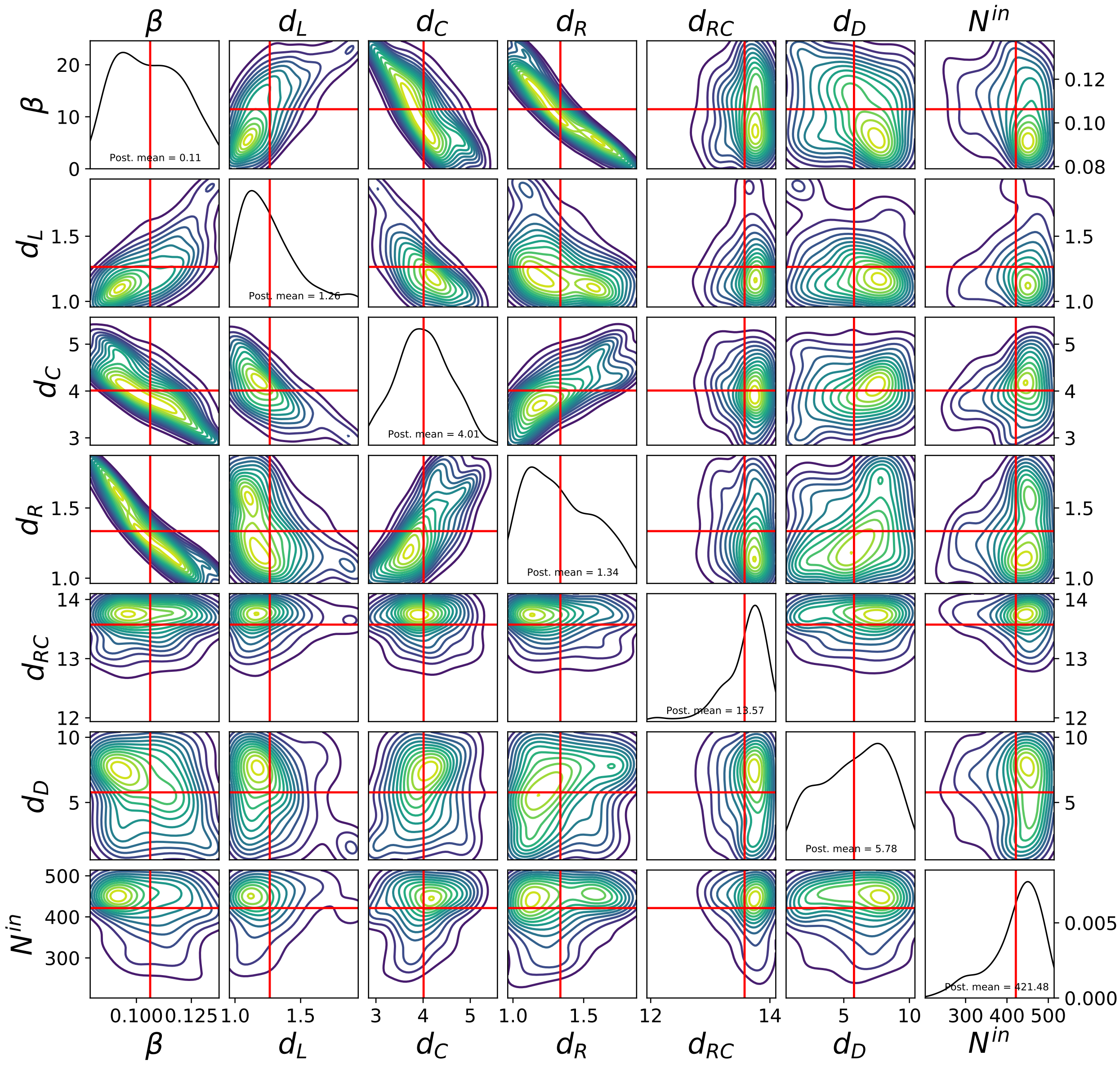

Figure 12: **Single and bivariate marginals (represented using contour plots) for the parameters describing the transition between states for France, obtained using data until 31st August**; note for instance the negative correlation between β and d_R; this is expected from the dynamics of the model (the longer a person stay in the infectious state, the more people can infect; therefore, a similar dynamics can be obtained by a lower value of β).

Table 2: **Estimated posterior mean and standard deviation of model parameters for France**. We point out that the estimated standard deviation is larger than the posterior mean for some of the parameters; this is due to the fact that the posterior distribution is not centered on the posterior mean but skewed.

**Observation period**	d_L	d_C	d_R	$d_{R,C}$	d_D
**1st March-31st Aug**	1.26 ± 0.22	4.01 ± 0.54	1.34 ± 0.23	13.57 ± 0.37	5.78 ± 2.54
**Observation period**	β	α_{123}	α_4	α_5	N^{in}
**1st March-31st Aug**	0.11 ± 0.01	0.31 ± 0.01	0.88 ± 0.10	0.19 ± 0.16	421 ± 61
**Observation period**	ρ_1	ρ_2	ρ_3	ρ_4	ρ_5
**1st March-31st Aug**	0.04 ± 0.04	0.03 ± 0.03	0.03 ± 0.03	0.91 ± 0.08	0.58 ± 0.08
**Observation period**	ρ'_1	ρ'_2	ρ'_3	ρ'_4	ρ'_5
**1st March-31st Aug**	0.38 ± 0.28	0.39 ± 0.29	0.39 ± 0.29	0.01 ± 0.01	0.58 ± 0.07

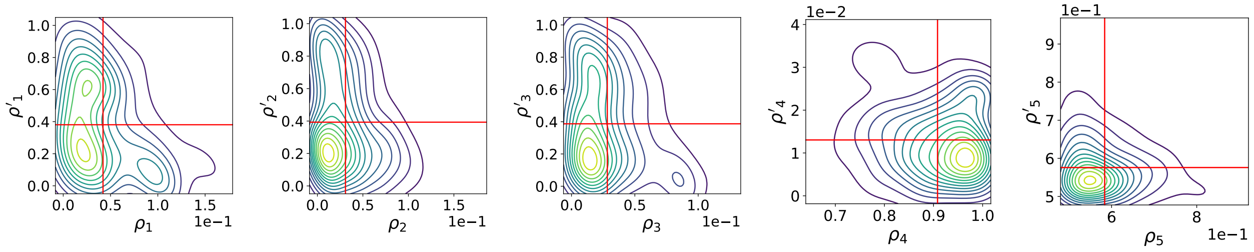

Figure 13: **Contour plots representing bivariate posterior plots for ρ_i and ρ'_i, for all age groups ($i = 1, \ldots, 5$) for France, obtained using data until 31st August**.

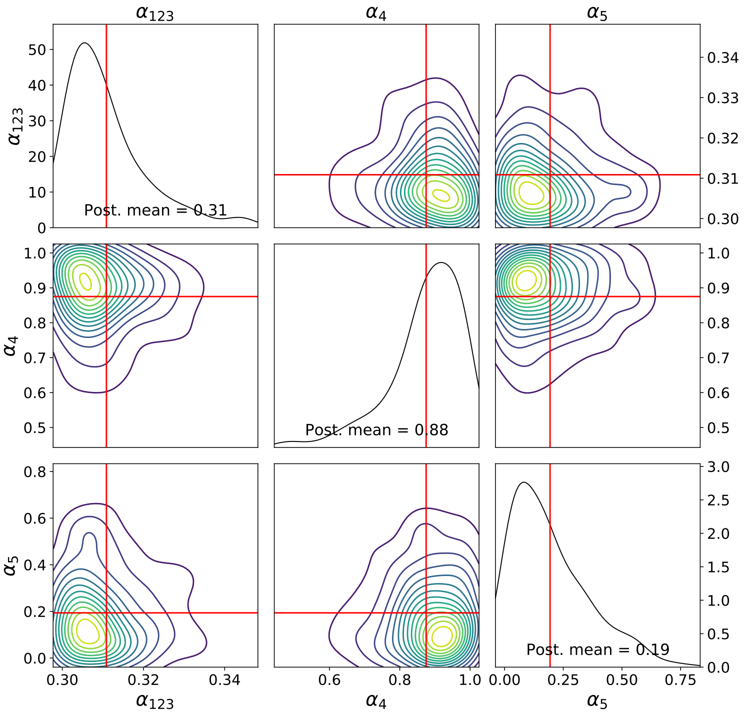

Figure 14: **Single and bivariate marginals (represented using contour plots) for the parameters connecting the reduction in mobility to the change of social contacts for France, obtained using data until 31st August**.

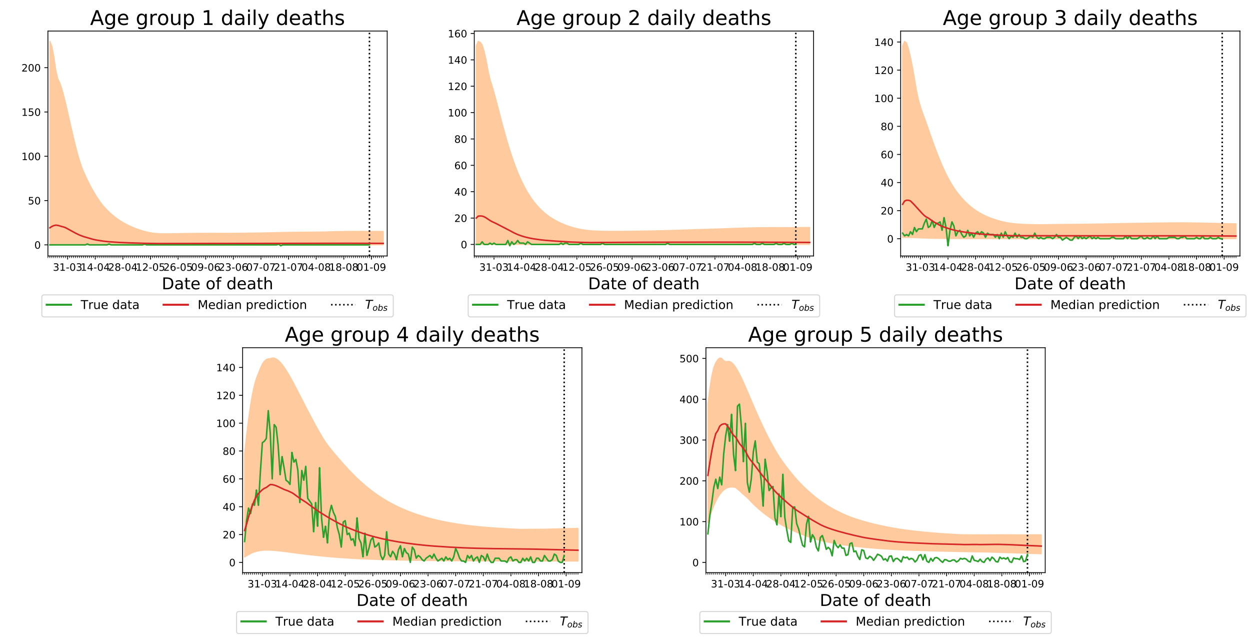

Figure 15: **Comparison of number of daily deceased predicted by our model and actual one (green line), for each age group for France, obtained using data until 31st August**.
