# Supplementary material for: Using mobility data in the design of optimal lockdown strategies for the COVID-19 pandemic
Source: PLoS Comput Biol. 2021 Aug 12;17(8):e1009236. doi: 10.1371/journal.pcbi.1009236 (PMC8360388; doi:10.1371/journal.pcbi.1009236)
Supplement: Attachment — Submitted filename: cover_resubmission.pdf [file pcbi.1009236.s003.pdf]

Coventry, UK, October 7, 2020

Dr. Ritabrata Dutta
Department of Statistics
University of Warwick

Editorial Board
PLOS Computational Biology

Dear Dr. Pitzer,

Thank you for your email on the 10th of August with comments on our manuscript ***"Using mobility data in the design of optimal lockdown strategies for the COVID-19 pandemic in England"*** (PCOMPBIOL-D-20-01162). We found your comments to be very useful and constructive, and we proceeded to make changes in the manuscript to incorporate your suggestions. We would now like to resubmit a revised version of our manuscript, where we took into account all the comments from the editor. Please find attached the reviewed manuscript, as well as a separate PDF file highlighting all the changes as requested. We also detail the main changes to the manuscript below. We first summarise the main major changes:

1. We changed the title of the paper: we removed "in England" from it, to highlight the fact that we can apply the methodology to any country which has the relevant data available. We have assessed the applicability of our approach by applying it to the French case, see point 4 below.

2. We renamed the methodology section to make our aims clearer and restructured the section to emphasize the connections between the different steps of the methodology and the mathematical and statistical tools used in each step.

3. In connection to point 1, we added Figures 1 and 5 to make our contributions more apparent. Figure 1 presents a flow diagram for the optimal lockdown procedure in a holistic manner, presenting the interconnection of all its different building blocks, from the priorities expressed by a policy maker, to the design of a bespoke lockdown strategy matching those demands. Figure 5 depicts another flow diagram which follows a similar philosophy, but focusing on the technical interplay between the assimilation of datasets and the computation of optimal lockdown strategies.

4. To highlight the applicability of our methodology outside of the UK, we applied our methodology to the evolution of COVID-19 in France and report on the corresponding sources of data where appropriate.

5. Moreover, we rewrote the Results and Discussion section. The section now starts with a summary of our results, and is then split into "proof of concept" of our methodology, where we test the sensitivity of our methodology to various "policy-maker" parameters – mainly relative weights of costs and optimisation windows, using the previously reported results - and application to the England and France datasets, with the models calibrated until the end of August and the lockdown strategies applied in September.

6. We removed the inference results from the Results and Discussion section and present these now in Appendix S2. We chose to do this in order to emphasise the optimal control part of our work which we believe to be the most relevant to report. We made the language in the Results and Discussion section less technical and hope that we have also achieved this by splitting this and the previous section in a more intuitive way. We tried to simplify the discussion to focus on the specific results and wrote it from the perspective of what we can and cannot achieve using our methodology. We also highlighted the places where input from a policy maker is essential. Finally, we also made the following minor changes:

7. We made minor adjustments to the introduction, adding some recent work and preparing for the upcoming changes in the following sections.

8. Similarly, we made a few minor changes in the conclusions, highlighting the advantages of our strategy compared to others, and reflecting on future work.

9. We added comments where appropriate, highlighting the applicability of the methodology to other applications (e.g., other epidemiological models (non-COVID), or other ODE based models) whenever the relevant data is available.

We hope that these changes address all your concerns, and that you find the new version appropriate for further review.

We look forward to hearing from you in due time.

Sincerely,

Dr. Ritabrata Dutta
on behalf of the authors
