# Supplementary material for: Using mobility data in the design of optimal lockdown strategies for the COVID-19 pandemic
Source: PLoS Comput Biol. 2021 Aug 12;17(8):e1009236. doi: 10.1371/journal.pcbi.1009236 (PMC8360388; doi:10.1371/journal.pcbi.1009236)
Supplement: Attachment — Submitted filename: PLOS.pdf [file pcbi.1009236.s004.pdf]

Using mobility data in the design of optimal lockdown strategies for the COVID-19 pandemic

Referee's Report:

The present paper deals with the development of mathematical modelling for the COVID-19 pandemic integrating mobility data in order to assess optimal lockdown policies.

The authors propose a data-driven control setup to tame the COVID-19 outbreak. Such methodology is divided in three main steps: statistical inferences of model parameters from public available data on Google mobility; a cost functional with the competition of economic loss and increase of the infection; numerical realization of the optimization procedure through NMPC methods.

The paper is well written and detailed, under many aspects: from the methodology aspects and the numerical experiments. I believe the results are interesting and worth of attention. I suggest publication after minor revision.

Find below my remarks.

- The model accounts contact rates based on age distribution. Is there any consistency condition that C_{ij} need to satisfy? In particular, since β is independent on the age I would expect a compatibility condition with respect to the evolution of the SEIRD over the sum of the age classes, e.g. $\beta \sum_i C_{ij} \simeq \beta$.
- Following the previous comment, the date rate ν does not depend on the class of age, is there any reason to not consider an age dependent death rate?
- How the model is initialized in terms of initial data? Or in other words how the Susceptible, S_i and Infected I_i are distributed in order to consider the demographic. I have seen this stated clearly.
- The cost functional (13) measures the impact of the infection. Why the expectation value is not accounted also for the basic reproduction number $\mathcal{R}(\cdot)$?
- Line 391, Formula (13) $\phi() \to \Phi()$.
