# Supplementary material for: Using mobility data in the design of optimal lockdown strategies for the COVID-19 pandemic
Source: PLoS Comput Biol. 2021 Aug 12;17(8):e1009236. doi: 10.1371/journal.pcbi.1009236 (PMC8360388; doi:10.1371/journal.pcbi.1009236)
Supplement: Attachment — Submitted filename: PCOMPBIOL-D-20-01162.pdf [file pcbi.1009236.s005.pdf]

Review report on the paper titled: *"Using mobility data in the design of optimal lockdown strategies for the COVID-19 pandemic"*.

The overall proposal is interesting and the revised version of the paper is more clear and complete with respect to the first draft. In the following there are some comments for the authors. The pages and lines refer to manuscript with the annotated corrections of the revision.

Specific comments

- In the abstract I suggest to delete the reference to the repository where the code is stored.
- As well described in the section of the paper titled *"Epidemiological model..."* the authors need to set up many assumptions to define the model dynamics: the contact matrices, the connections between mobility and contacts, the initializations of the compartments and the definition of the cost functionals. The fact that the proposal requires so many assumptions which are often untestable should be well stressed in the introduction as well as in the conclusion of the paper.
- Page 3 line 79 add some reference to introduce the ABC method also considering the following reference: Brown, G. D., Porter, A. T., Oleson, J. J., Hinman, J. A. (2018). Approximate Bayesian Computation for spatial SEIR(S) epidemic models. Spatial and Spatio-temporal *Epidemiology*, 24: 27–37.
- Page 4 text of Fig 1: check if the use of the sign plus within the words can be avoided.
- In section titled *"Epidemiological model..."* you write many times the words "our" and "our model", it would be preferable to reduce these repetitions. Check the whole paper.

- Page 5 specify the abbreviation for "ODEs".
- Page 26: clarify the meaning of the sentence " we assume the once of England are well represented by those".
- Page 26 line 176: the symbol * used in the paper is misleading, for example the notation at page 10 "$* \in$" is not usual. Another notation can be for example $\tilde{p}$.
- Page 26 Fig. 4 add "we" also before the other "note".
- Equation at Page 12: delete the first parenthesis that is not necessary.
- Page 14: delete the reference to the Python library since it is also stated in the S1 Appendix.
- Page 18, lines 617, 617: justify better the fact that the proposal of a prediction horizon of 30 day is aligned with the COVID-19 time scale for transmission, adding a proper reference.
- Page 20: please check when you refer to Fig. 13: reference is correct? What about the credibility intervals? I could not find Figures 16 and 17 in the manuscript.
- At the end of the section where the results for England and France are present would be useful to add some general considerations on the estimated difference which can help the reader to understand the reasons of the corresponding strategies for each country.
- Table 1 in the S1 Appendix: when you introduce the table recall section of the paper where you define the parameters. Check that all the parameters are well defined in the paper and add the proper reference in appendices S1 and S2.
- Some of the considerations made at Section 1.2 of the S2 Appendix are important and they have to be added in the concluding remarks of the paper. I mainly refer for what concerns the mentioned difficulty to forecast precisely the evolution of the epidemic, the uncertainty connected with the choice of the time horizon and the overestimation of the number of deceased in the tail of the epidemic.
