# Supplementary material for: Using mobility data in the design of optimal lockdown strategies for the COVID-19 pandemic
Source: PLoS Comput Biol. 2021 Aug 12;17(8):e1009236. doi: 10.1371/journal.pcbi.1009236 (PMC8360388; doi:10.1371/journal.pcbi.1009236)
Supplement: Attachment — Submitted filename: Response_Reviewers_Comments.pdf [file pcbi.1009236.s006.pdf]

Coventry, UK, February 17, 2021

Dr. Ritabrata Dutta
Department of Statistics
University of Warwick

Editorial Board
PLOS Computational Biology

Dear Dr. Pitzer and Dr. Ferrari,

Thank you for your email on the 10th of February with reviewers' comments on our manuscript ***"Using mobility data in the design of optimal lockdown strategies for the COVID-19 pandemic"*** (PCOMPBIOL-D-20-01162). We found the comments to be very useful and constructive, and we proceeded to make changes in the manuscript to incorporate the suggestions. Below we address in detail each reviewer's comments.

Moreover, we attach the reviewed manuscript, as well as a separate PDF file highlighting all the changes as requested.

Comments by Reviewer 1

- The model accounts contact rates based on age distribution. Is there any consistency condition that C_{ij} need to satisfy? In particular, since β is independent on the age I would expect a compatibility condition P with respect to the evolution of the SEIRD over the sum of the age classes, e.g. $\beta \sum_i C_{ij} \simeq \beta$.

  The contact matrices are estimated from studies on a sample of the population of the given country, and then corrected in order to account for the total population size in each each category (see for instance the discussion in Section 3 in [?]); as such, their definition already embeds some consistency constraints given the population size in a given age category. Specifically, as C_{ij} represents the mean daily number of contacts an individual in age group i has with individuals in age group j, and given that the contacts are reciprocal, the contact matrices are defined so that $n_i \cdot C_{ij} = n_j \cdot C_{ji}$, where n_i is the population size in age group i.

  Further, we remark that β in our model represents a contact specific probability of transmission; this is different from, for instance, the definition of the non-age splitted SIR model (as given e.g., here), in which notation

beta represents the product of the number of contacts and the probability that each of them results in an infection. The difference in our model comes from explicitly factoring the age-specific contact matrix C_{ij}. For this reason, there is no compatibility condition that the sum of elements in C_{ij} needs to satisfy with beta, as they represent two independent model elements (contacts and probability of infection). We note that we assume that each contact (no matter the age groups involved) has the same probability of resulting in an infection.

- Following the previous comment, the date rate ν does not depend on the class of age, is there any reason to not consider an age dependent death rate?

  We agree that we could have made ν to be age dependent; however, we had already inserted age dependence in the two transition probabilities ρ and ρ'; further adding age dependence in ν would lead to more expensive parameter estimation, without certainty in gain of predictive power of the model

- How the model is initialized in terms of initial data? Or in other words how the Susceptible, S_i and Infected I_i are distributed in order to consider the demographic. I have seen this stated clearly.

  We point that the initialization of the model dynamics using age distribution of the International travellers and described between Lines 222-238.

- The cost functional (13) measures the impact of the infection. Why the expectation value is not accounted also for the basic reproduction number $\mathcal{R}(\cdot)$?

  In fact there was an error in the statement of Eq. (13); in our implementation, we estimated the expectation of $\mathcal{R}(\cdot)$; we corrected the equation.

- Line 391, Formula (13) $\phi() \to \Phi()$.

  We modified the text accordingly.

Comments by Reviewer 2

- In the abstract I suggest to delete the reference to the repository where the code is stored.

  We moved this to Conclusion.

- As well described in the section of the paper titled "Epidemiological model..." the authors need to set up many assumptions to define the model dynamics: the contact matrices, the connections between mobility and contacts, the initializations of the compartments and the definition of the cost functionals. The fact that the proposal requires so many assumptions which are often untestable should be well stressed in the introduction as well as in the conclusion of the paper.

  Added this line 'We would like to stress here that, in order to define the model dynamics, we have considered some assumptions (on the contact

matrices, the connection between mobility data and contacts, the initialisation of the compartments and the definition of the cost functional) which may not be easily tested in real a life scenario.' at the end of Introduction.

- Page 3 line 79 add some reference to introduce the ABC method also considering the following reference: Brown, G. D., Porter, A. T., Oleson, J. J., Hinman, J. A. (2018). Approximate Bayesian Computation for spatial SEIR(S) epidemic models. Spatial and Spatio-temporal Epidemiology, 24: 27–37.

  We modified the text accordingly.

- Page 4 text of Fig 1: check if the use of the sign plus within the words can be Avoided.

  Updated from 'data + dynamics updated model' to 'Inferred Dynamical model'

- In section titled "Epidemiological model..." you write many times the words "our" and "our model", it would be preferable to reduce these repetitions. Check the whole paper.

  We modified the text accordingly.

- Page 5 specify the abbreviation for "ODEs".

  We modified the text accordingly.

- Page 26: clarify the meaning of the sentence " we assume the once of England are well represented by those".

  We modify it to 'We assume that the contact matrix for England to be the same as that for the whole of the UK.'

- Page 26 line 176: the symbol $*$ used in the paper is misleading, for example the notation at page 10 "$* \in$" is not usual. Another notation can be for example $\tilde{p}$

  We modified the text to a more conventional notation.

- Page 26 Fig. 4 add "we" also before the other "note".

  We modified the text accordingly.

- Equation at Page 12: delete the first parenthesis that is not necessary.

  We modified the text accordingly.

- Page 14: delete the reference to the Python library since it is also stated in the S1 Appendix.

  We removed the reference to Scipy.

- Page 18, lines 617, 617: justify better the fact that the proposal of a prediction horizon of 30 day is aligned with the COVID-19 time scale for transmission, adding a proper reference.

  PHE now considers a COVID-related death as a death which happened within 28 days of a positive test (see PHE reporting of COVID-19 deaths: technical summary – 12 August 2020) Hence, we believe that a 30 days window is sufficient to account for this.

- Page 20: please check when you refer to Fig. 13: reference is correct? What about the credibility intervals? I could not find Figures 16 and 17 in the manuscript.

  This confusion is probably due to the reviewer reading the wrong annotated version. We have ensured that there is no discrepancy present in Figure 13 and we do not have any Figure 16/17 in the manuscript and we do not refer to them.

- At the end of the section where the results for England and France are present would be useful to add some general considerations on the estimated difference which can help the reader to understand the reasons of the corresponding strategies for each Country.

  We add in the manuscript 'We can see a significant difference in the data reported in these two countries (green lines in Figure 12) which leads to very different parameter estimates of the epidemic model (see S2 Appendix). This is reflected in the optimal control we inferred for these two countries, highlighting the adaptability of our methodology for different countries and different scenarios.' However we are not in a position to provide a public healthcare perspective explaining this difference between the number of people hospitalized and death in these two countries.

- Table 1 in the S1 Appendix: when you introduce the table recall section of the paper where you define the parameters. Check that all the parameters are well defined in the paper and add the proper reference in appendices S1 and S2.

  We modified the text accordingly.

- Some of the considerations made at Section 1.2 of the S2 Appendix are important and they have to be added in the concluding remarks of the paper. I mainly refer for what concerns the mentioned difficulty to forecast precisely the evolution of the epidemic, the uncertainty connected with the choice of the time horizon and the overestimation of the number of deceased in the tail of the epidemic.

  We remarked this in the conclusion section.

We hope that these changes address all the points raised by the reviewers concerns, and that you find the new version appropriate for publication.

We look forward to hearing from you in due time.

Sincerely,

Dr. Ritabrata Dutta
on behalf of the authors

References

[1] Klepac P, Kucharski AJ, Conlan AJ, Kissler S, Tang M, Fry H, et al. Contacts in context: large-scale setting-specific social mixing matrices from the BBC Pandemic project. medRxiv. 2020;.
